# Ligand binding and dynamics of the monomeric epidermal growth factor receptor ectodomain

**DOI:** 10.1002/prot.24339

**Published:** 2013-08-19

**Authors:** Hannes H Loeffler, Martyn D Winn

**Affiliations:** Scientific Computing Department, STFC DaresburyWarrington, WA4 4AD, United Kingdom

**Keywords:** molecular dynamics, free energy, molecular mechanics Poisson–Boltzmann surface area, extracellular domain, receptor–ligand, Her1, ErbB1

## Abstract

The ectodomain of the human epidermal growth factor receptor (hEGFR) controls input to several cell signalling networks via binding with extracellular growth factors. To gain insight into the dynamics and ligand binding of the ectodomain, the hEGFR monomer was subjected to molecular dynamics simulation. The monomer was found to be substantially more flexible than the ectodomain dimer studied previously. Simulations where the endogeneous ligand EGF binds to either Subdomain I or Subdomain III, or where hEGFR is unbound, show significant differences in dynamics. The molecular mechanics Poisson–Boltzmann surface area method has been used to derive relative free energies of ligand binding, and we find that the ligand is capable of binding either subdomain with a slight preference for III. Alanine-scanning calculations for the effect of selected ligand mutants on binding reproduce the trends of affinity measurements. Taken together, these results emphasize the possible role of the ectodomain monomer in the initial step of ligand binding, and add details to the static picture obtained from crystal structures. Proteins 2013; 81:1931–1943. © 2013 The Authors. Proteins published by Wiley Periodicals, Inc.

## INTRODUCTION

The human epidermal growth factor receptor (hEGFR), also referred to as ErbB1 or Her1, is a receptor tyrosine kinase (RTK) and part of a family of four closely related receptors (ErbB1–ErbB4). These RTKs provide input to a complicated signalling network controlling such vital cell functions as proliferation, migration, differentiation, and apoptosis. Their activity is controlled by a set of 13 extracellular ligands which bind to the ectodomain.^1^ Some ligands bind to more than one family member but none are known to bind to ErbB2. These RTKs are expressed in many kinds of tissues including skin, internal organs, breast, and prostate, and dysfunction in the network may lead to serious illnesses, in particular various types of cancers. Consequently, the ErbB network is one of the most studied systems of signal transduction.

The hEGFR monomer comprises an ectodomain with four subdomains (I–IV), a single transmembrane helix, the juxtamembrane region, a tyrosine kinase domain, and the C-terminal regulatory region. The active receptor is usually considered to be the dimer, with both homo and hetero dimers occurring within the ErbB family. Structural information comes from crystallography of individual domains,^2^–^6^ Nuclear magentic resonance (NMR) as well as infra-red and circular dichroism (CD) spectroscopy of the transmembrane helices and the juxtamembrane domain,^7^–^9^ small angle X-ray scattering (SAXS) of soluble ectodomains,^10^ and negative-stain electron microscopy.^11^–^13^ From crystallography, the ectodomain of the hEGFR monomer is known to exist either in an autoinhibited (so-called tethered) conformation^2^–^4^ or in an open conformation.^5^,^6^ The latter is capable of dimerising and thus helping to form the active receptor. The existence of these two conformations in solution has been confirmed by SAXS.^10^ Negative-stain electron microscopy has also suggested distinct densities consistent with different ectodomain conformations.^13^

The exact mechanism of ligand binding is unknown but in principal two models are possible.^14^ One model assumes that a small fraction of the unbound monomer exists in the open conformation and ligand binding to that conformation drives the thermodynamic equilibrium to the side of the dimer.^2^ The other model requires the ligand to bind first to the tethered conformation to initiate opening and subsequent dimerization. One crystal structure of the tethered hEGFR monomer does in fact include an epidermal growth factor (EGF) ligand bound to subdomain I, but this is not considered to be biologically relevant.^2^ Scatchard analysis of ligand binding indicates two populations of binding sites, and this has been explained in terms of negative cooperativity of ligand binding to the receptor dimer.^15^ A possible structural basis for this negative cooperativity has been proposed based on crystal structures of Drosophila EGFR,^16^ and from simulations of human EGFR ectodomains on the plasma membrane.^17^ The parameterized model of MacDonald and Pike^15^ implies significant dimerization of unliganded ectodomain dimers, which would require ligand-independent conformational changes of the monomer.

Molecular dynamics (MD) simulations provide a means to supplement experimental structural data with atomic-level information about protein dynamics. Such information is vital to understanding a flexible multiconformation protein such as hEGFR. In recent years, several MD studies of the ectodomain dimer^17^–^22^ and of the tyrosine kinase domain^21^,^23^–^28^ have emerged. The hEGFR tethered monomer has not yet been the subject of MD simulations, although there is a recent study of the closely related ErbB4 monomer^29^ whose dynamics and ligand-binding properties are less well-understood. An understanding of the tethered monomer would help clarify the earliest steps of ligand-induced activation of hEGRF.

In addition to exploring the protein dynamics, simulation can also help to understand the energetics of ligand binding. Numerous methods have been devised to estimate free energies of binding Δ*G*_bind_ of biologically relevant ligands to proteins.^30^ Some approaches attempt to obtain Δ*G*_bind_ through direct simulation of the binding event, but such methods are typically computationally expensive as many intermediate states need to be explicitly enumerated and/or multiple runs are required to achieve sufficient numerical convergence. The ectodomain consist of the first 614 residues of hEGFR, whereas the EGF ligand has 53 residues, and so even the monomeric receptor-ligand system is computationally challenging. Some end-point methods on the other hand may conveniently be carried out after the MD trajectory has been computed. Necessarily, these approaches are less reliable in terms of accuracy and absolute binding energies may not be quantitatively possible, but relative energies and ranking may be within reach.^31^,^32^ One such method is the molecular mechanics Poisson–Boltzmann surface area (MM–PBSA) approach.^33^–^35^ With MM–PBSA, coordinates from the trajectory are post-processed to evaluate gas-phase force field energies (the MM part) and to estimate the free energy of solvation through solution of the Poisson–Boltzman equation (the PB part) and an expression relating to the surface area (the SA part). Δ*G*_bind_ can then be estimated from combining these contributions computed for complex, receptor, and ligand.

A large number of site-directed mutagenesis experiments on ligands have been carried out to gain a deeper understanding of the ligand's interactions with the receptor and the functional role of individual residues. Relevant to the current study are mutations of the EGF ligand at Leu47,^36^,^37^ Arg41,^38^,^39^ Lys28,^40^ and Ile23.^37^,^40^–^42^ To relate these experiments to structural models of ligand binding, a computational technique called “alanine scanning” can be used. In silico mutations of residues to alanine are analyzed with the MM–PBSA method to obtain relative free energies of binding for such ligand variants.

In this study, we investigate the dynamics and ligand binding of the monomeric hEGFR ectodomain via MD simulation. Experimental insights into the structural transitions of the monomer have come from crystal structures,^2^–^4^ from SAXS measurements,^10^ and from tryptophan fluorescence.^14^. Here, we use MD simulations and the MM–PBSA method to investigate a structural model of the monomeric ectodomain, and relate our results to those experiments. A picture emerges of a highly flexible protein, which complicates simple models for ligand binding.

The work here lays the foundation for understanding ligand binding in other members of the ErbB family, and in the yet larger dimeric system. It may help to rationalize the effects of dimer asymmetry on ligand binding, seen in Drosophila EGFR^16^ and in simulations of hEGFR on the plasma membrane.^17^

## MATERIALS AND METHODS

### Simulation setup

Three independent simulations of the hEGFR ectodomain in the tethered conformation were carried out with: (1) the EGF ligand bound to domain I (bI), (2) the EGF ligand bound to domain III (bIII), and (3) the ligand removed from the receptor (ub). The starting structure for bI was taken from PDBid 1NQL,^2^ which is a crystal structure of the monomeric tethered hEGFR ectodomain with EGF bound to domain I. To construct the configuration where EGF is attached to domain III, we fitted domain III of PDBid 1IVO^5^ to 1NQL and transferred the ligand coordinates. The unbound simulation was prepared by removing EGF from 1NQL.

Protonation states of titratable residues at pH 7 were determined with PROPKA^43^,^44^ using the PDB2PQR web interface.^45^ The histidines in the domain I binding site, His23, and the domain III binding site, His346, His359, and His409, were all δ-protonated in each of the three simulations. These structures were subjected to 500 steps of initial minimization with heavy atoms restrained. The proteins were immersed in a cubic water box and counter ions were added to create an approximately 0.1*M* solution of NaCl. Thus, 177 Cl^−^ and 183 Na^+^ ions were added for the bI, 177 Cl^−^ and 184 Na^+^ ions were added for the bIII, and 180 Cl^−^ and 182 Na^+^ ions were added for the ub simulations, respectively. The total number of atoms was about 395,000 for each simulation system leaving a water buffer of at least 30 Å between the protein and the closest box face.

All heavy atoms of the receptor and the ligand were subjected to positional restraints of 5 kcal/mol/Å. Each system was energy minimized for 1000 steps to relax atom positions and remove bad contacts from the setup procedure. Next, the simulation boxes were heated incrementally from 25 K to the final temperature of 300 K in steps of 1000 over a period of 10,000 steps and kept at the final temperature for an additional 10,000 steps. Over a further 25,000 steps a constant pressure of 1 bar was applied to adjust the box size and the density. The temperature was controlled with a Langevin thermostat and a coupling coefficient of 1 ps^−1^. The pressure was controlled by a Nosé–Hoover Langevin barostat^46^,^47^ with a decay time of 1000 fs and an oscillation period of 2000 fs. Finally, restraints were switched off step-wise over a period of 10,000 steps in increments of 1000 steps for initial preparation of the production run.

The total unrestrained simulation times were 150 ns for each of the three systems. All MD simulations were carried out at a constant temperature of 300 K and a constant pressure of 1 bar. The Langevin barostat decay time was set to 100 fs and the oscillation period to 200 fs. The simulations were run with NAMD^48^ in versions 2.6 and 2.7. The force fields used were CHARMM 22 for proteins^49^ with torsional backbone corrections (CMAP^50^) and ions,^51^ and TIP3P for water.^49^,^52^ All bonds to hydrogens were constrained with the SHAKE algorithm which allowed for a time step of 2 fs. This simulation protocol is essentially the same as in our previous studies.^17^,^22^

### Binding free energies

Binding free energies were estimated by means of the MM–PBSA method. 33,34,53 MM–PBSA is an endpoint method that allows convenient post analysis of the trajectory or trajectories. The binding free energy Δ*G* is calculated from the individual free energies of the three species as (1)
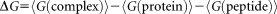
where complex is the hEGFR monomer plus the EGF ligand (bound to either Domain I or Domain III), protein is hEGFR and peptide is EGF. Each free energy *G* is computed as an average over the trajectory in the following way: (2)
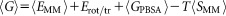
where *E*_MM_ is the molecular mechanical energy for the chosen force field including internal and nonbonding degrees of freedom, *G*_PBSA_ is the solvation free energy with the polar contributions computed through the Poisson–Boltzmann equation and the nonpolar part estimated from the scaled and shifted molecular surface, *T* is the simulation temperature, and *S*_MM_ is the conformational entropy of the solute.*E*_rot/tr_ is the energy due to the six rotational and translational degrees of freedom and in the classical limit is 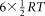 (=1.79 kcal/mol at 300 K).^35^ In the computation of a relative free binding energy ΔΔ*G*, this term cancels, and is often neglected.

In principle, three independent simulations must be carried out to calculate each term in Eq. 1. In some situations, it may be possible to obtain a reasonable estimate for Δ*G* by sampling all three species from a single simulation of the complex. The assumption is that the conformations sampled for protein and peptide in the complex are representative for the unbound state. In the next section, we will discuss how far this is true for the systems studied here.

The MM–PBSA calculations have been carried out with CHARMM^54^,^55^ 35 using a script developed by us.^56^ We validated our implementation by computing Δ*G* for the Ras-Raf complex (a standard test system), and comparing to the Amber results (see Supporting Information). The force field energies *E*_MM_ were calculated without nonbonded cutoffs. The nonpolar contributions to *G*_PBSA_ were evaluated with CHARMM's surface module by computing them as *G*_nonpolar_ = γ*A* + β 57,55 where *γ* and *β* were 0.00542 kcal/Å^2^ and 0.92 kcal/mol, respectively, and *A* is the molecular surface in Å^2^ computed with the atomic radii by Nina *et al*.^58^ The polar contributions were calculated with APBS^59^ using the CHARMM 22 partial charges and a grid resolution of 0.3 Å (see Supporting Information) which required up to 13 GB of memory. Snapshots were taken every 100 ps (1400 data points) from the bIII simulation and every 200 ps (700 data points) from the bI and ub simulations. The trajectory averages were then calculated omitting the first 10 ns of simulation. For the three-trajectory approach, a 75 ns reference simulation of the EGF ligand was used, taking snapshots every 100ps (650 data points). Standard errors were estimated using the approach detailed in Supporting Information.

## RESULTS

### Dynamics of the bound and unbound receptor ectodomain

#### Flexibility

The root mean square deviations (RMSD) of C_α_ atoms were calculated relative to the structures from the initial minimization step for the four subdomains of the receptor ectodomain. For each simulation, the largest deviations are found for Subdomain II (see Supporting Information, Fig. S1) associated with a flexing along the length of this subdomain. This is in contrast to the soluble ectodomain dimer in which Subdomain II is stabilized by the dimer interface, and Subdomain IV is more flexible.^22^

**Figure 1:**
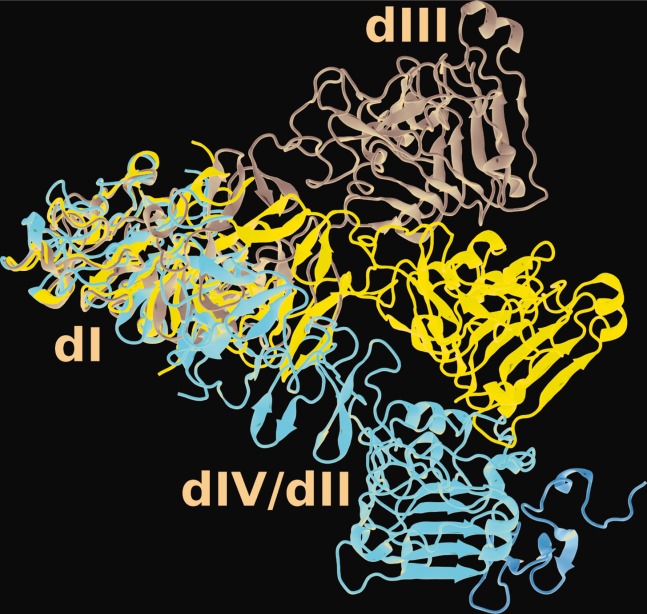
The bIII simulation structure (red, ligand in orange bound to domain III) taken from a snapshot at 106.27 ns, compared to the crystal structures 1NQL (grey, ligand removed) and 1YY9 (blue, antibody removed) aligned on residues 6–156 (domain I). Domain I is on the left, Domains II and IV in front, and Domain III on the right. [Color figure can be viewed in the online issue, which is available at wileyonlinelibrary.com]

Relative motions of the subdomains were analyzed by computing RMSDs with respect to a fixed subdomain I (see Supporting Information, Fig. S2). Although Subdomain II maintains its position relative to Subdomain I, Subdomains III and IV fluctuate strongly, and usually in a correlated manner. The largest fluctuation is seen for the bIII simulation with a relative displacement of 70 Å reached after 106 ns of the simulation. This snapshot is compared in Figure 1 to the crystal structure from which the starting configuration was taken (1NQL^2^), and a second crystal structure (1YY9^3^).

**Figure 2:**
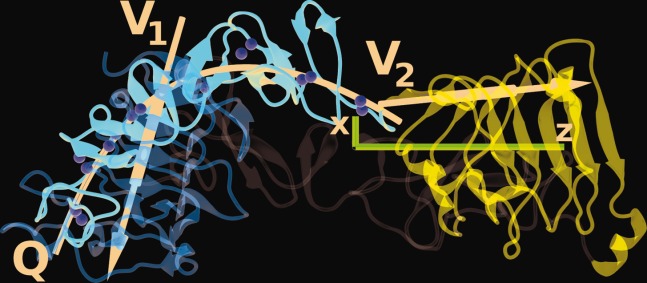
Definition of vectors *V*_1_ and *V*_2_ used to define the orientations of Subdomains I and III. Q denotes the quadratic fit curve through the center of mass of the disulfide modules of subdomain II. Sulfur atoms of disulfide bridges are shown as yellow spheres. The magenta coordinate system shows the eigenvectors (scaled by a factor of 1/20) of the *R*_g_ tensor with *z* the longest axis and *x* the shortest. Domain I (orange, top) is closest to the viewer and on top of domain II (red). Domain IV (grey) is farthest away. Domain III is shown in blue. [Color figure can be viewed in the online issue, which is available at wileyonlinelibrary.com]

Analysis of RMSDs suggests a highly flexible hEGFR monomer, with Subdomains III and IV moving relative to Subdomain I, at least partly driven by flexing of Subdomain II. To obtain a clearer picture, we have computed angle distributions between two vectors *V*_1_ and *V*_2_ defining the orientations of Subdomains I and III (see Fig. 2). As reference points for these vectors, we chose the C_α_ atoms of residues Val36 and Glu118 in Subdomain I and the “equivalent” residues Ser340 and Glu431 in Subdomain III. As these residues are located at the ends of a relatively stable parallel β sheet and both domains are quite rigid (see Supporting Information, Fig. S1), effects of intramolecular motion are kept to a minimum.

**Figure 3:**
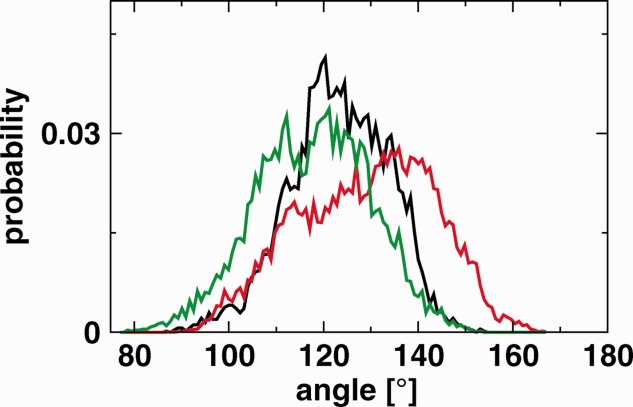
Angle distribution between the vectors *V*_1_ and *V*_2_ (for definitions see Fig. 2) for the monomeric simulations bI (black), bIII (red), and ub (green). All snapshots for each trajectory have been included in the calculation. [Color figure can be viewed in the online issue, which is available at wileyonlinelibrary.com]

As Figure 3 shows, the monomer simulations exhibit a broad distribution of angles between Subdomains I and III highlighting the large displacements that take place. Although there is considerable overlap in the distributions, there are clear differences between the three simulations. For comparison, the three available crystal structures of the tethered ectodomain show angles of 98.5° (1NQL), 113.0° (3QWQ^4^), and 130.1° (1YY9). Simulation bIII, with the EGF ligand bound to Domain III of the receptor, tends to larger angles than the other simulations, and larger than seen in any crystal structure.

We have also calculated the maximum curvature of a quadratic function fitted through the center of masses of the seven disulfide bond modules of Subdomain II following the procedure of Du *et al*.^29^ This serves as a measure for the flexing of the “spine–like” structure^60^ of the domain (see illustration in Fig. 2).

**Figure 4:**
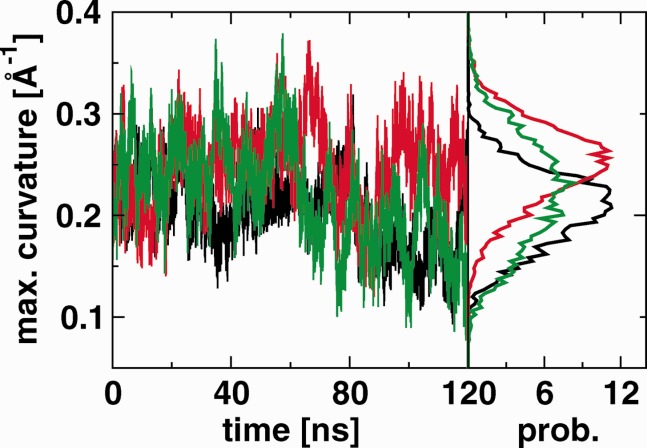
Maximum curvature of the quadratic fit curve Q (see Fig. 2) for the monomer simulations bI (black), bIII (red), and ub (green). The time series are shown on the left, and the resulting distribution on the right. [Color figure can be viewed in the online issue, which is available at wileyonlinelibrary.com]

Figure 4 depicts the time series and the resulting distribution functions of the maximum curvatures from the three monomer simulations. The curvature of Subdomain II is consistently higher in the bIII simulation than in the bI simulation, and the distribution functions show clearly separated peaks (maxima at 0.21 Å^−1^ for bI and at 0.25 Å^−1^ for bIII). The unbound monomer shows a wider fluctuation of the curvature, and the distribution covers the range of the two other peaks. From the simulation of the soluble ectodomain dimer,^22^ the subdomain II curvatures of the constituent monomers peak at 0.28 Å^−1^ and 0.34 Å^−1^. For comparison, the three available crystal structures of the tethered ectodomain show maximum curvatures of 0.36 Å^−1^ (1NQL), 0.36 Å^−1^ (3QWQ), and 0.19 Å^−1^ (1YY9).

#### Changes in shape

**Figure 5:**
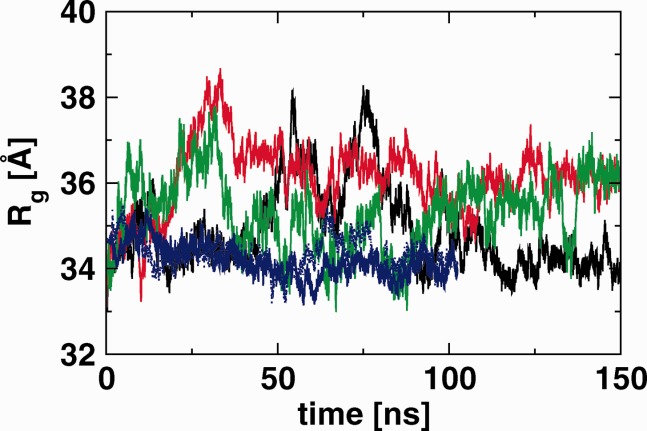
Radius of gyration *R*_g_ as a function of time for the monomeric simulations bI (black), bIII (red), ub (green), and for both monomers of the dimeric hEGFR simulation (solid and dotted blue). The ligand has not been included in the calculations. [Color figure can be viewed in the online issue, which is available at wileyonlinelibrary.com]

For each simulation, the gyration tensor has been calculated for the receptor (i.e., excluding the ligand atoms) as a function of simulation time. These tensors are highly anisotropic, reflecting the nonglobular shape of the ectodomain monomer; the average ratios of the eigenvalues are 833/311/76 Å^2^ for bI, 898/318/71 Å for bIII, and 875/302/71 Å for ub. The largest eigenvalue lies along the main axis of the molecule, and an example is shown in Figure 2. To compare with SAXS experiments, it is useful to compute the equivalent scalar radius of gyration *R*_g_. Time courses of *R*_g_ are presented in Figure 5, and average values over the last 120 ns are summarized in Table1. The graphs show considerable fluctuations, with variations of up to 4 Å in the case of the bI simulation.

**Table 1:** Radius of gyration *R*_g_ and maximum dimension *D*_max_ in Å as obtained from MD simulation and experiment.^10^

	Simulation^a^		Experiment^b^	
	R_g_	D_max_	R_g_	D_max_
bI	35.0	126.3		
bIII	35.9	126.9		
Ub	35.2	125.4	35.4 ± 0.11	105 ± 5
Her1–Her1, monomer 1	34.1	139.0		
Her1–Her1, monomer 2	34.3	142.4		
Her1–Her1, dimer	42.3	142.4	46.1 ± 0.89	145 ± 5

a No carbohydrates.

b Fully glycosylated proteins.

The ligand has been excluded.

The average *R*_g_ compares well with the experimental value of 35.4 ± 0.11 Å obtained from SAXS.^10^. However, that value was obtained from a fully glycosylated protein, whereas the model used in the current study is not glycosylated. Calculations on the crystal structure 1NQL have shown that model sugars (Man_9_GlcNAc_2_) attached to known and presumed glycosylation sites can add about 3 Å to the *R*_g_ when positioned approximately perpendicular on the molecular surface.^10^ Adding model sugars to the current MD simulations would likely lead to a smaller increase due to relaxation of the oligosaccharides. Furthermore, the oligosaccharides would be expected to interact with each other, with the protein surface, and with neighbouring receptor domains. These interactions may have a significant effect on the overall shape.

The *R*_g_ values computed for the individual monomers in the dimer simulation^22^ (with ligands excluded) are smaller than the values for the tethered monomers, with an average of around 34.2 Å. The average ratios of the eigenvalues of the gyration tensor are 840/244/85 Å^2^ and 859/234/87 Å^2^. Thus, the monomers in the dimer complex are more compact on average, despite being in the “extended” conformation. The latter is, however, reflected in the larger value for the maximum dimension *D*_max_.

**Figure 6:**
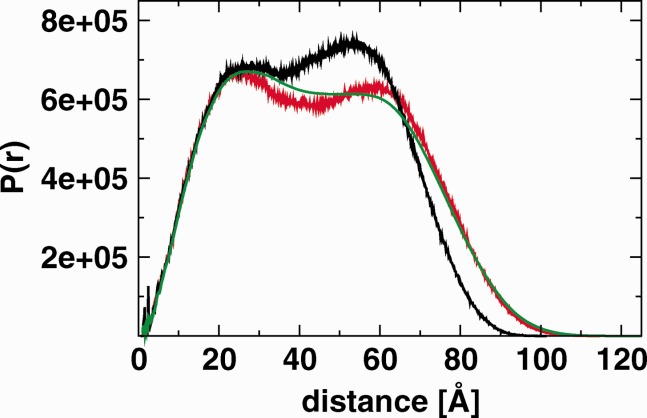
Pair distribution function *P*(r) for the monomeric simulation ub (green) compared with data obtained from the receptor component of the X-ray structures 1NQL (black) and 1YY9 (red). [Color figure can be viewed in the online issue, which is available at wileyonlinelibrary.com]

Figure 6 compares the average pair distribution function *P*(r) obtained from the simulation of the unbound monomer (last 120 ns) with curves calculated directly from the receptor component of the crystal structures 1NQL^2^ and 1YY9.^3^ 1NQL was used to construct the starting structures for the simulations, whereas 1YY9 (which is in complex with a Fab fragment) has a significantly different conformation (see Fig. 1). The simulation curve encompasses both the short-distance peak of 1NQL and the large-distance tail of the less compact 1YY9. Thus, although the simulation was started from a particular crystal structure, it has explored different conformations, including ones similar to that captured in a different crystal structure. The differences between the three simulations (Supporting Information, Fig. S4) are relatively smaller, confirming that each system shows this flexibility.

#### Principal component analysis

Principal component analysis (PCA) has been carried out to gain further insights into the dynamics of the three hEGFR simulations. As large proteins are notoriously slow to converge;^61^ however, we applied this method here only to reveal differences between the dynamics of the three simulations. The mutual scalar products of the first five eigenvectors (Supporting Information, Tables SI and SII) show that with further progress of the simulations the individual modes become less similar, meaning that the three simulations diverge and lead to different global motions.

Projecting the trajectory against the first two PCA modes (Supporting Information, Fig. S7) for each simulation further confirms different dynamics in the three systems. For example, the bI simulation displays possible energy barriers in the 2D trajectory, while phase space is populated more densely in the other simulations. However, the probabilities obtained from the projections of the trajectory onto the first five eigenvectors (Supporting Information, Fig. S6) indicate strong non-Gaussian distributions in the lowest modes. The largest amplitude motions cannot be expected to be harmonic but the rather broad distribution suggests sampling issues. It would therefore be misleading to attach too much significance to the motions implied by the PCA modes.

### Binding free energies of the ligand

The binding free energy Δ*G* of the ligand EGF to its monomeric receptor hEGFR has been estimated with the MM–PBSA method.^33^,^34^,^53^ We are particularly interested in the ΔΔ*G* between EGF bound to Domain I and EGF bound to Domain III. This may suggest where (if at all) the ligand preferentially binds to the monomeric receptor, as well as identify the relative contributions of the two interfaces in the ligated dimer. Absolute Δ*G*s are difficult to obtain^35^,^62^,^63^ especially in the case of the large complexes discussed here.

The crystal structure 1NQL was found to bind the ligand weakly at domain I at pH 5.^2^ The interactions of the EGF–Domain I interface primarily involve backbone hydrogen bonds which are not expected to be affected by changes in pH. In contrast, the EGF–Domain III interface may be disrupted at lower pH because of protonation of three histidine side chains in Domain III (see Simulation Setup). At neutral pH, a Domain III fragment was found to bind EGF strongly with a *K*_d_ of about 450 nm.^64^ Binding to the full-length receptor is similar in strength, but falls off at low pH as Domain III binding is disrupted.^2^ The residual Domain I binding is therefore presumed to be weak, and the binding seen in 1NQL to be an artifact of the protein concentrations used for crystallization.

In Table2, we summarize computed binding free energies obtained with both the single trajectory and three trajectories approaches (see Methods). The table also shows a test of the grid resolution used in the Poisson–Boltzmann calculation for the polar part of the solvation free energy.Δ*G* is sensitive to the grid resolution used, with a finer grid leading to a more negative Δ*G* in most cases. Although there is no obvious convergence with respect to the grid resolution, finer grids lead to smaller fluctuations over the trajectory (see Supporting Information, Fig. S8) and are assumed to be more accurate.

**Table 2:** Free energies of binding of EGF to Domain I or Domain III of the receptor obtained with the MM–PBSA method.

	Res^a^	Domain I		Domain III	
Single		Three	Single	Three
Δ*G*_noS_^b^	0.75	−62.4	−52.4	−52.4	−78.9
Δ*G*_noS_	0.5	−76.8	−70.4	−61.2	−96.7
Δ*G*_ele_	0.3	−222.8 ± 45.2	−400.0 ± 80.9	−105.4 ± 29.9	−272.5 ± 75.8
Δ*E*_vdW_		−69.1 ± 5.5	−106.7 ± 7.6	−61.2 ± 6.6	−68.1 ± 8.0
Δ*E*_int_		0.0	28.3 ± 7.5	0.0	3.7 ± 7.0
Δ*E*_MM_		−291.9 ± 49.0	−478.5 ± 83.2	−166.6 ± 34.2	−336.8 ± 78.6
Δ*G*_PB_		222.4 ± 38.9	417.2 ± 74.6	107.8 ± 31.8	258.3 ± 72.6
Δ*G*_SA_		−12.1 ± 0.6	−14.8 ± 0.8	−10.6 ± 0.3	−8.7 ± 0.7
Δ*G*_noS_		−81.6 ± 16.5	−76.1 ± 13.2	−69.3 ± 9.4	−87.3 ± 9.4
−TΔ*S*_rt_^c^			30.9		30.7
−TΔ*S*_v_^d^			8.5		11.6
−TΔ*S*_tot_^e^			39.3		42.3
Δ*G*_tot_^f^			−36.8		−45.0

a Grid resolution in PB calculation in Å.

b Δ*G*_noS_ = *E*_MM_ + Δ*G*_PB_ + Δ*G*_SA_.

c Rotational plus translational entropy.

d Vibrational entropy from normal mode analysis.

e Δ*S*_tot_ = Δ*S*_rt_ + Δ*S*_v_.

f Δ*G*_tot_ = Δ*G*_noS_ – *T*Δ*S*_tot_ based on 0.3 Å resolution.

Results from both the single trajectory and three trajectories approach are presented. Energies are in kcal/mol.

Comparing the single with the three trajectories approach, we find that they give very different Δ*G* values. Moreover, the two approaches suggest opposite binding preferences, with the ligand preferring Domain I with the single-trajectory approach and Domain III with the three trajectories approach. The high flexibility of the ectodomain and the differences seen between the bound and unbound simulations indicate that representative receptor or ligand conformations cannot realistically be taken from the trajectory of the complex. This can be quantified with the reorganization free energy^65^,^66^ Δ*G*_reorg, noS_ which is the free energy difference of a particular component in the complex and in an unbound state. We find (ignoring the conformational entropy) 5.9 and 12.3 kcal/mol for the ligand, and −0.4 and −30.2 kcal/mol for the receptor in the bI and bIII simulations, respectively. We thus conclude that the single-trajectory approach is not valid for the system considered here. Finally, Δ*G*s were calculated only for the three trajectories approach.

The vibrational entropy Δ*S*_v_ has been estimated through normal mode analysis. However, obtaining entropies from the very large hEGFR–ligand complex (≅ 10,000 atoms) is not only very time and memory consuming but it is also difficult to calculate reliable minimized structures. Therefore, we followed a previous suggestion^67^ to work on a reduced system. Normal modes are calculated for a flexible region consisting of the ligand and all receptor residues within 8 Å of the ligand. A buffer region with fixed atoms prevents distortions of the flexible region during energy minimization, and avoids problems of uncapped residues. Further details on the definition of the regions and the entropy calculation are given in Supporting Information.

As shown in Table2, Δ*S*_v_ is similar in magnitude for both Domain I and Domain III binding. As the ligand is also in close contact to Domains II and III in the Domain I binding case, we included also parts of Domain II and III (residues 261–375) in the entropy calculation and computed a new Δ*S*_v_ which was almost the same (8.8 kcal/mol) as in the smaller system (8.5 kcal/mol). We conclude that the change in vibrational entropy has only a minor effect on the final Δ*G*_tot_. In many MM–PBSA studies, it is in fact neglected from the outset (see e.g., Refs. 27 and 68).

The final result for Δ*G*_tot_ predicts that domain III binding is more likely than Domain I binding by about 8 kcal/mol. The statistical uncertainty in Δ*G*_noS_, however, is slightly larger than the final ΔΔ*G* of binding, and so the preference is not clear cut.

Table2 also summarizes component energies contributing to the free energy of binding. In general, the magnitudes of the components are larger in the three trajectories approach than they are in the single-trajectory approach, suggesting that the assumption behind the single-trajectory approach is suppressing some energetic changes. For both approaches, the gas-phase binding energy Δ*E*_MM_ favors complex formation with a preference for Domain I binding over Domain III binding. This is driven by more favorable changes in the electrostatic energy Δ*E*_ele_, and to a lesser extent in the van der Waals energy Δ*E*_vdW_. In contrast, solvation contributions to the free energy change, and in particular the Poisson–Boltzmann contributions Δ*G*_PB_, oppose complex formation but are less unfavorable for Domain III binding. The overall free energy change for ligand binding is the result of a competition between gas-phase energetics and solvation effects. In the three trajectories approach, the latter effect is stronger, and Δ*G*_tot_ favors binding of EGF to Domain III.

#### Ligand mutations

In Table3, we summarize binding free energies for four single-point mutations of the EGF ligand, plus a null mutation included as a control. Leu47 and Arg41 are involved in Domain III binding, and are conserved across five hEGFR ligands: EGF, TGFα, betacellulin, epiregulin, and heparin-binding EGF-like growth factor. Lys28 and Ile23 are involved in Domain I binding; Lys28 is conserved in TGFα only, whereas Ile23 is replaced by similar side chains (Leu and Val) in other hEGFR ligands. Results were obtained through the alanine-scanning method in which a single residue is mutated into an alanine, but protein conformations are taken from the original simulations.^33^ The alanine-scanning method applied here has the limitation that it assumes conformations of the mutant are identical to those of the wild-type, that is, no new simulation is run. In general, NMR and binding replacement data find only small localized changes to the mutant–receptor interactions with no significant changes to the fold of the ligand,^36^,^38^ but this might not always be the case.^69^

**Table 3:** Free energies of binding for selected mutations computed via alanine-scanning.

	L47A		R41A		K28A		I23A		A25A	
	dI	dIII	dI	dIII	dI	dIII	dI	dIII	dI	dIII
Δ*E*_ele_	−400.0	−272.5	−382.6	−180.0	−376.7	−246.8	−400.1	−272.5	−400.0	−272.5
Δ*E*_vdW_	−107.0	−63.5	−105.8	−67.7	−106.6	−68.0	−104.2	−68.0	−106.7	−68.1
Δ*E*_int_	28.2	3.7	27.8	4.3	27.1	3.9	28.2	3.8	28.2	3.6
Δ*E*_MM_	−478.8	−332.3	−460.6	−243.2	−456.2	−310.9	−476.2	−336.7	−478.5	−337.0
Δ*E*_PB_	417.2	257.7	400.0	173.0	395.1	233.7	417.0	258.1	417.2	258.3
Δ*E*_SA_	−14.8	−8.4	−14.7	−8.5	−14.6	−8.7	−14.8	−8.7	−14.8	−8.7
Δ*E*_noS_	−76.4	−83.0	−75.3	−78.7	−75.7	−85.9	−74.0	−87.3	−76.1	−87.4
Δ*E*_noS_	−0.3	4.3	0.9	8.6	0.4	1.4	2.1	0.0	0.0	−0.1
Expt.		2.5^36^		0.05^38^,^39^		79–95^a^	3^41^	—		
		2^37^		180–188^b^						

a Relative binding affinity for mutation K28R.^40^

b Relative binding affinity for mutation K28L.^40^

The contribution of the conformational entropy is not included. All results are for the three trajectories approach. ΔΔ*G*_nos_ gives the change with respect to the wild-type free energy of binding. Energies are in kcal/mol. Experimental relative binding affinities = IC_5_0(wild type)/IC_5_0(mutant) are in %.

The L47A and R41A mutations have little effect on Domain I binding, but are unfavorable for Domain III binding as expected. Comparing the component energies with those of the wild-type (Table2), the difference for L47A comes largely from a change in Δ*E*_vdW_, whereas the difference for R41A comes from large (and partially compensating) changes in Δ*E*_ele_ and Δ*G*_PB_. The I23A mutation is close to the wild type in Domain III binding, but Δ*G*_noS_ is slightly less favorable for Domain I binding. As for L47A, this difference comes largely from a change in Δ*E*_vdw_. The K28A mutation has only a small effect on both Domain I and Domain III binding. As for R41A, the electrostatic contributions vary strongly from the wild type, but in this case largely cancel out.

It must be noted that the ΔΔ*G*_noS_ values calculated for these mutants are smaller than the standard errors of the individual Δ*G*_noS_. On the other hand, we are reassured that the changes in the individual components make chemical sense, and that the control calculation for A25A gives essentially zero (not necessarily precisely zero because the alanine-scanning protocol rebuilds side chains in an idealized position and conformation).

## DISCUSSION

Molecular simulation of the tethered hEGFR monomer provides a detailed picture of the dynamics of both the unbound state (ub) and bound states where the EGF ligand binds to either domain I (bI) or domain III (bIII). In all three cases, the monomer remains in the tethered conformation for the duration of the simulation. Thus, any major structural rearrangement is likely to occur on a longer timescale than the 150 ns probed by our atomistic simulation. This is in agreement with a recent simulation study of the ErbB4 ectodomain monomer, with the ligand NRG1β bound to Domain I, which showed a transition to a partially extended state at around 400 ns after breaking of the Domain II/Domain IV tether.^29^ Recent experimental studies of tether mutants^10^ and a truncated form of ErbB4 missing Domain IV^70^ show that the tether is not required to maintain a tether-like conformation, suggesting that there are several stabilizing interactions to be overcome.^10^

Nevertheless, our simulations do reveal that the tethered monomer is extremely flexible as demonstrated by, for example, angle (Fig. 3) and maximum curvature (Fig. 4) distributions. Correlated motions of Domain III and IV with respect to Domains I and II are clearly visible in RMSDs (see Supporting Information, Fig. S2). The largest deviations are seen in bIII, where the structure may move away from the starting structure by as much as 70 Å and fluctuations regularly span 30 Å. These motions are in the same overall direction as revealed by the difference between the two crystal structures 1NQL^2^ and 1YY9^3^ (see Fig. 1). Li *et al*.^3^ suggested that these two structures “provide two snapshots of a flexible domain II, which have been trapped by different crystal packing environments,” and our simulation supports this interpretation. A recent crystal structure of the tethered monomer in complex with an adnectin^4^ shows a third relative position of Domain III, intermediate between 1NQL and 1YY9.

Dimerization has a strong effect on both intradomain and interdomain motions generally leading to a globally less dynamic system. In previous work,^17^,^22^ we have discussed the flexibility of the ectodomain dimer, in particular when placed in a membrane environment. Although such flexibility is significant, and may be relevant for ligand affinity in the dimer, the flexibility seen in the current study for the monomer is much larger.

From a study of tryptophan fluorescence, Kozer *et al*.^14^ found evidence for multiple conformations of the unliganded monomer of a truncated hEGFR ectodomain, as well as a rotational correlation time that was shorter than that for a rigid monomer. In contrast, in the presence of ligand there was only a single conformation with a rotational correlation time appropriate to a rigid complex. They interpreted this in terms of an equilibrium between tethered and open conformations in the absence of ligand, with ligand-binding stabilizing the open conformation (here, we use “tethered” as a convenient label, although their truncated form of hEGFR precluded the formation of the Domain II/Domain IV tether). Our simulation results suggest an alternative interpretation. Even in the tethered conformation, the unbound monomer shows considerable flexibility, with changes in the relative orientation between Domains I and III (see Fig. 3), and this may be sufficient to explain the experimental results. The ligand bound dimer is certainly less flexible, and the same is probably true of the ligand-bound extended monomer.

A central question for growth factor binding is whether these ligands bind to the tethered conformation, and if so whether there is a preference for the Domain I or the Domain III binding site. In their simulation of the tethered ErbB4 ectodomain, Du *et al*.^29^ conclude that the ligand neuregulin-1β preferentially binds to Domain I, based on a trial simulation where the ligand moves to Domain I from a starting position between Domains I and III. We note that such a simulation is likely to be highly dependent on the starting position and orientation of the ligand. Furthermore, it is known that the Domain III ligand binding site is rotated 130° away from the Domain I binding site in going from the extended to the tethered conformation (see Ref.^2^ for hEGFR or compare 2AHX^60^ and 3U7U^71^ for ErbB4). Thus, the ligand cannot be orientated so as to potentially satisfy both binding surfaces.

We therefore consider both Domain I and Domain III binding to remain potentially relevant. Ligand binding in both systems (bI and bIII) appears stable over the 150 ns of simulation. Nevertheless, we also see clear differences between these simulations, suggesting that the location of ligand binding has an effect on the dynamics of the monomer. Figure 3 shows a difference in the interdomain angle distribution of bIII, compared to the bI and ub simulations. Although the change in the relative orientation of domain III is not in the direction required to form the extended conformation, it does suggest that ligand binding to Domain III induces a change in conformation which may be relevant, whereas (according to this measure) no such change occurs upon binding to Domain I. Correlated motions of Domains III and IV are also in general larger when the ligand is bound to Domain III (see Supporting Information, Fig. S2).

Conversely, binding of the ligand to Domain I leads to larger fluctuations in Domain II than when the ligand binds to Domain III (see Supporting Information, Fig. S1). Du *et al*.^29^ highlight Domain II bending during the first stage of their simulation (in which the neuregulin-1β ligand is bound to Domain I), suggesting that a large fluctuation eventually leads to the breaking of the tether. On the other hand, the distribution of the maximum curvature of Domain II (see Fig. 4) shows larger curvatures when the ligand is bound to Domain III, and so the mechanism proposed by Du *et al*. may be applicable in this case as well.

Application of the MM–PBSA method gives some insight into the free energy of ligand binding. The highly dynamical hEGFR monomers rule out the use of the single-trajectory approach in which it is assumed that representative conformations for all three of complex, receptor, and ligand can be obtained from a single simulation of the complex. The large conformational changes seen over tens of ns also support the use of relatively long simulation times (i.e., 150 ns). An analysis of the correlation between snapshots used in the MM–PBSA calculation shows correlation times up to 10 ns for some components. For comparison, Luo *et al*.^72^ performed similar calculations for ErbB3 and ErbB4 complexes taking snapshots from 1 ns simulations, whereas Fuentes *et al*.^68^ applied MM–GBSA to complexes of ErbB2 with antibodies taking snapshots from 20 ns simulations. Although the latter studies were applied to the more rigid extended conformation, it is clear that longer simulations times are necessary to get adequate statistics for the tethered conformation.

Both Domain I and Domain III of hEGFR contribute to ligand binding in the extended conformation.^5^,^6^ In contrast, if the ligand binds to the tethered conformation, then it can only form an interface with one of the domains. There may be an energetic preference, and the binding free energies estimated here from the three trajectories approach suggest that the EGF ligand binds more favorably to Domain III than to Domain I. Loosely, Domain I binding is driven by gas-phase electrostatic interactions, whereas Domain III binding is driven by solvation terms, and overall the latter is predicted to be the larger effect (Table2). However, the difference in binding free energy for bI and bIII is comparable to the associated standard errors and is perhaps not significant. Because Domain III binding is driven by solvation effects, its relative strength will be affected by changes in the solvent composition and this may be another factor behind the observed preference for Domain I in the crystal structure.^2^

In a recent comparison of different EGFR ligands, Sanders *et al*.^20^ find a Δ*G*_bind_ for binding of EGF to dimeric EGFR of −102 kcal/mol. Entropy terms are not included, so this is to be compared to the values of −76 and −87 kcal/mol taken from Table2 for binding to Domain I and Domain III, respectively. A comparison hints that binding to the extended conformation is more favorable, but that the contributions of Domains I and III are not simply additive.

The changes in binding free energy we have found for selected ligand mutants (Table3) are mostly in qualitative agreement with experiment.^36^–^42^ Experimentally, the largest reductions in binding affinity have been found for mutants of Arg41, with R41A having 0.05% of the affinity of the wild type (determined as the ratio of *IC*_50_ values).^38^,^39^ The affinity of L47A is reduced to 2^37^ and 2.5% 36 of the wild-type, whereas the affinity of I23A is reduced to 5.9%. 41 The relative order of these three mutants is the same as in our calculations (see Table3). A K28A mutant has not been reported in the literature, but the affinity of a K28L mutant was 79% of the wild-type, whereas the K28R variant was 188%. 40 Our calculation showing the K28A mutant to have only a minor effect on binding is consistent with these results, and with the lack of conservation of Lys28 among hEGFR ligands.

**Figure 7:**
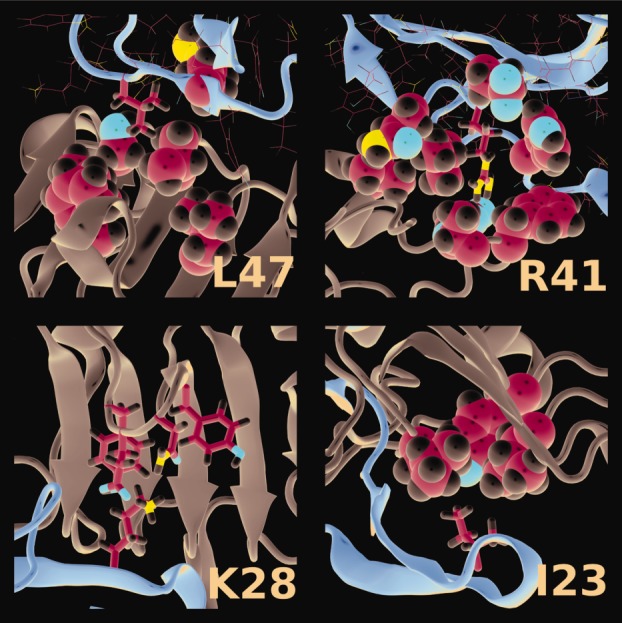
The interactions of the residues Leu47, Arg41, Lys28, and Ile23 with their immediate environment. Key residues are highlighted. Leu47: Val417, I438, S440 in direct contact with Leu, Phe412, and Leu382 flanking this region, Arg45 from the ligand; Arg41: salt bridge to Asp355, near neighbors Val350, Phe357, Thr358, and Tyr13, Leu15, Glu40, Gln43 from the ligand; Lys28: Tyr89, Glu90, Asn91, Tyr93; Ile23: Leu14, Leu69, Tyr45. hEGFR backbone in grey and EGF backbone in brown.

Figure 7 shows zoomed snapshots of residues Leu47, Arg41, Lys28, and Ile23, together with their key interactions. In the simulation, Arg41 of EGF forms a strong salt bridge to Asp355, as is observed in the crystal structure.^5^ In the alanine variant, this interaction is removed and the aspartic acid is exposed to a mostly hydrophobic tightly packed environment (Val350, Phe357 and Thr358, and Leu15 from the ligand). This is consistent with the guanidinium group being an absolute requirement for high affinity.^39^ In our simulations, as in the crystal structure,^5^ Leu47 is embedded in a hydrophobic pocket (Leu382, Phe412, Val417, Ser440, Ile438, Ile467). The mutation to alanine causes a disfavorable change in the van der Waals interactions as the smaller residue packs less well into the pocket. Despite breaking the strong salt bridge to Glu90 on Domain I of the receptor in the K28A variant, the predicted effect on domain I binding affinity is marginal. Measuring the heavy atom distance (CD–NZ) for that salt bridge we find that this distance is smaller than 4.0 Å in 91% of the total simulation time, which might suggest that the salt bridge does contribute to the binding. In contrast, if we look at the same salt bridge in our previous simulations of the liganded-dimer,^17^,^22^ then we find that it is often broken in the symmetric dimer in solution, and almost always broken in the asymmetric dimer on the membrane. We conclude that this salt bridge is in fact weak. Finally, Ile23 is in close contact to Leu14, Leu69, and Tyr45 in Domain I of the receptor, the latter making a hydrogen bond to the backbone of Leu14. Mutation to alanine will reduce the van der Waals interactions, but the calculated effect on Domain I binding is quite small.

Our results for key mutations thus make some sense in terms of the known structural interactions. They confirm the experimental affinity measurements and suggest that specific ligand interactions with Domain III are vital for ligand binding. Although ligand binding to Domain I is predicted to have a similar strength overall, partly due to backbone hydrogen bonding, specific side chain interactions appear to be less important.

In general, the current results may be limited by the simplicity of the structural model. First, we have not modeled the membrane environment, in contrast to our earlier study^22^ where it played a crucial role. Second, we have not modeled the oligosaccharide chains which can decorate the protein at up to 12 N-linked glycosylation sites.^73^,^74^ It has been argued that disruption of the steric restraints due to the presence of oligosaccharides may be one factor required to break the tether.^10^

In summary, our calculations suggest that the EGF ligand can bind to the tethered monomer, and that binding has an effect on the dynamics of this flexible protein. There are clear differences between Domain I and Domain III binding of the ligand, with some hints that Domain III binding produces more significant changes in the average conformation. Free energy estimates also show a slight preference for ligand binding to Domain III. Although we find that ligand binding to Domain I is also possible, the evidence of a role for specific ligand residues is not so strong. These two binding interfaces are of course also relevant to ligand binding in the extended dimer, where both contribute to the overall binding. If asymmetric dimers^16^,^17^ occur, then the relative contributions of the two binding interfaces could vary.
