# Supplementary material for: Ligand binding and dynamics of the monomeric epidermal growth factor receptor ectodomain
Source: Proteins. 2013 Aug 19;81(11):1931–43. doi: 10.1002/prot.24339 (PMC4282322; doi:10.1002/prot.24339)
Supplement: Supplementary file 1 — Supporting Information [file prot0081-1931-SD1.pdf]

Ligand Binding and Dynamics of the Monomeric Epidermal Growth Factor Receptor Ectodomain (Supplement)

Hannes H. Loeffler[*], Martyn D. Winn

April 25, 2013

1 Deviations from the reference crystal structure

The structural stability of the hEGFR ectodomain monomer and its two possible complexes with EGF has been analyzed via the root mean square deviation (RMSD) of C_α atoms calculated relative to the structures from the initial hydrogen minimization step (see Methods). The receptor component of these reference structures is essentially that of the crystal structure 1NQL[2]. We note that the crystal structure was obtained at pH 5.0 while the simulation here assumed a pH of 7.0. The RMSDs of the three simulations with EGF bound to domain I (bI), EGF bound to domain III (bIII) and the unbound receptor (ub), together with RMSDs for the soluble hEGFR dimer obtained in our previous study[22], are shown in Figure S1. The RMSDs are calculated separately for each subdomain.

The smallest RMSDs were generally observed for domain I and domain III, as expected for these relatively rigid subdomains. The domain I RMSD is smaller than the domain III RMSD in the bIII and ub simulations, but slightly larger in the bI simulation which may be a consequence of EGF binding. The RMSDs of those two domains also show a slight upwards drift from about 1.5 Å to about 2 Å in the bI simulation. The domain IV RMSD overlaps to a high degree with the domain I and III RMSDs but exhibits larger fluctuations. The largest deviations are seen for domain II, in particular for the bI simulation with values reaching up to 7 Å. This large intra-domain flexibility is associated with a bending along the length of domain II. Previously, in the context of asymmetric dimer structures[16,17], a flexible region around Asp238 in domain II has been identified. In the monomer simulations, the flexibility is distributed over a wide residue range, including Asp238 but with significant contributions from residues at the C-terminal end of domain II.

There is a certain degree of correlation between the domain II and domain IV RMSDs although it appears weaker in the bIII simulation. This correlation is not surprising as both domains remained tightly tethered together. Strong hydrogen bonds were found between Tyr246–N and Met576–O, and Tyr246–OH and Asp563–OD2. There is also a weak H–bond between Tyr246–OH and Lys585–NZ. These hydrogen bonds were observed in the crystal structure[2]. In contrast, side chain H–bonds were not found for the doubly protonated His566, or between Asn247 and Glu578. These H–bond patterns were essentially the same in all three simulations.

[*]Hannes.Loeffler@stfc.ac.uk

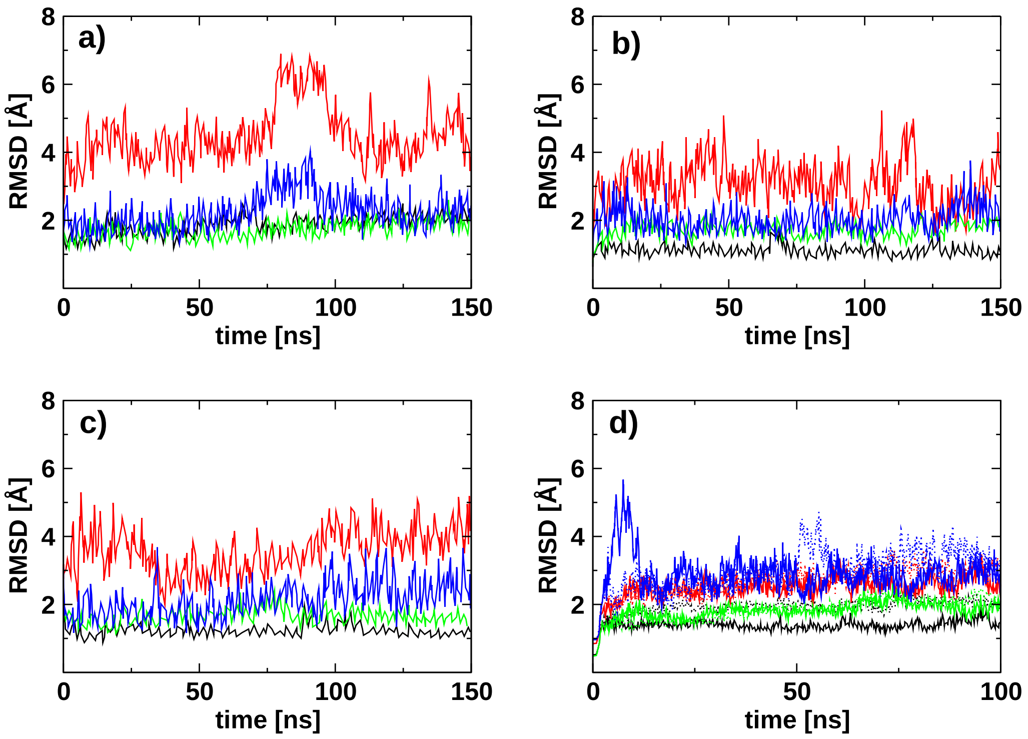

Figure S1: Root mean square deviations of the C_α atoms for the three monomeric simulations a) ligand bound to domain I, b) ligand bound to domain III, c) unbound, and d) the 100 ns simulation of the hEGFR dimer[22]. The colors are black for domain I, red for domain II, green for domain III, and blue for domain IV. The dotted lines in d) are for the second monomer.

Comparing with the hEGFR ectodomain dimer simulation, the most obvious difference is that the domain II RMSD is smaller and the domain IV RMSD is somewhat larger than in the monomeric simulations. This is consistent with the stabilizing influence of the dimerisation interface on domain II, and the loss of the stabilizing tether for domain IV. Domain I and III RMSDs are very similar in both the monomeric and dimeric simulations.

To investigate the relative movement of domains during each simulation, we have computed RMSDs with respect to a fixed domain I. The RMSDs in Figure S2 show how far domains II, III, and IV have moved away from domain I in 150 ns of simulation.

In the monomeric simulations, domain III moves furthest away from its initial position, by up to 70 Å in the case of the bIII simulation. The wide range of RMSD values sampled by domains III and IV underlines the high flexibility of the monomers. Domain II generally stays within a region of 5–10 Å. Occasional returns to a structure close to the reference structure were also observed.

Correlated movements of domains III and IV are clearly visible. In the case of the bI simulation the two domains sample very similar RMSDs over wide portions of the simulation. A similar effect is visible in the bIII simulation but with the domain IV RMSD generally smaller than the domain III RMSD. This difference in RMSDs was more pronounced in the ub simulation. Movements of domains III and IV also show some correlation with the intra-domain flexibility of domain II (see Figure S1), and it is likely that the inter-domain motions are partly driven by bending of domain II (see maximum curvature calculations in main text).

The dimer simulation displays much smaller RMSDs, with similar values for the domains II and III in both monomers. Domain IV moves farthest away from its initial position and fluctuates the most, which demonstrates the higher flexibility of this domain as discussed previ-

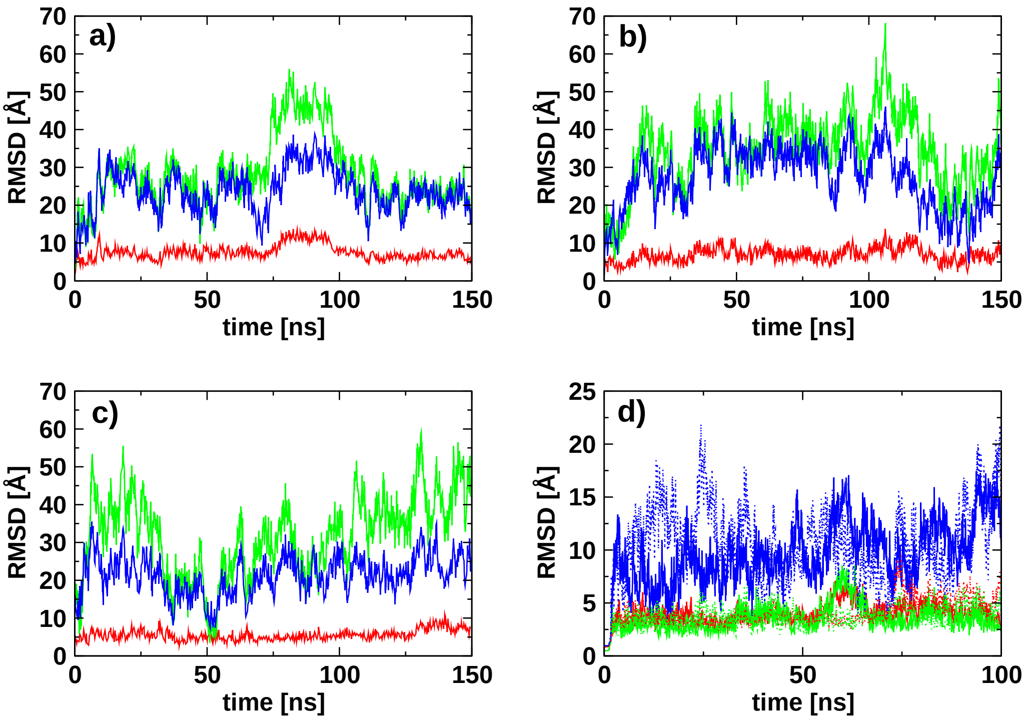

Figure S2: Root mean square deviations of the C_α atoms relative to fixed domain I for the three monomeric simulations a) ligand bound to domain I, b) ligand bound to domain III, c) unbound, and d) the 100 ns simulation of the hEGFR dimer[22]. The colors are red for domain II, green for domain III, and blue for domain IV. The dotted lines in d) are for the second monomer. Note the different scale in the y–axis in the dimer simulation.

ously[22]. All fixed RMSDs have been calculated with respect to domain I of the same monomer.

In summary, the receptor monomers show much greater flexibility than the core of the dimer (i.e. excepting the flexible domains IV of the latter). Domain II shows the greatest intra-domain flexibility, while interdomain flexibility is characterized by movement of domains III/IV with respect to domains I/II. Within this general picture, there are some differences depending on whether the receptor binds the ligand on domain I, domain III or not at all.

2 Pair distribution functions

Small angle X-ray scattering (SAXS) can be used to probe the spatial extent of molecules as the radius of gyration and the pair distribution function are readily obtained. Figure S3 summarises various contributions to the overall pair distribution function P(r). The P(r) has been calculated for the bI simulation (Her1 with ligand bound to domain I) excluding the ligand and for time steps between 30 and 150 ns. Figure S3a) depicts contributions for the individual intra-domain P(r) for domains I to IV. As expected their peaks are located at short distances, about 20 Å for domains I and III, and about 15 Å for the two other domains. Domains II and IV have widely distributed pair distances with a long tail extending well beyond 60 Å. In figure S3b) P(r) contributions are computed for a single domain with respect to all other domains, e.g. domain I (red) vs. domains II, III, and IV. Domain II peaks at 37 Å, domain IV at 54 Å and both domains I and III at 60 Å. Figures S3c) and d) show contributions for one domain vs. one other domain. Domain I is closest to domain II (peak at 33 Å) and nearly equally far from both domains III and IV (59 and 61 Å, respectively). Domains II and III pair distances are distributed

over a wide range of distances. The P(r) between the domain I/II pair and the domain III/IV pair is illustrated in figure S3e). This distribution displays a strong peak at 60 Å and thus dominates the second peak in the total P(r) but as figure S3d shows some of the contributions also come from the domain III–IV P(r). The absence of this peak in the extended conformation correlates with this finding.

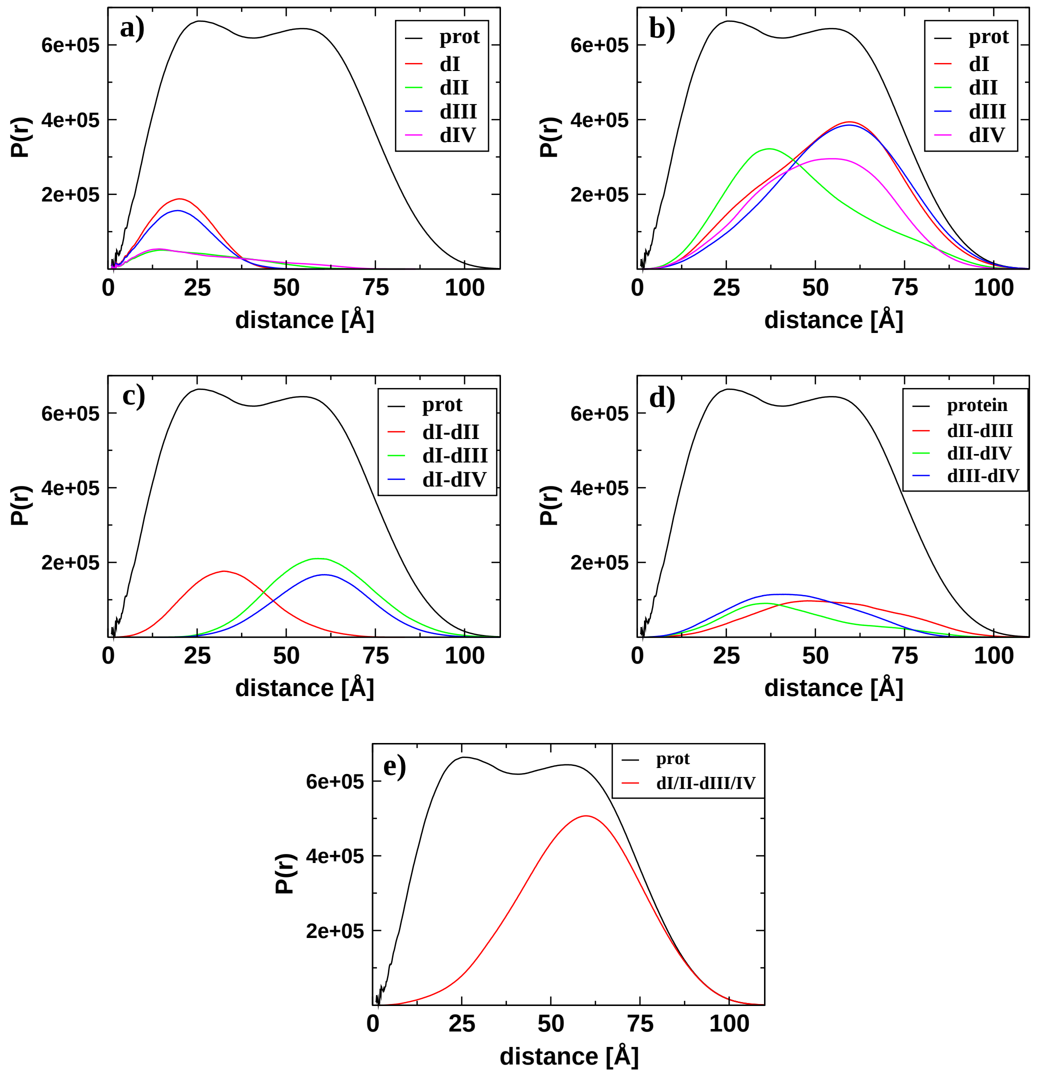

Figure S3: Subdomain contributions to the overall P(r) for the bI simulation (Her1 with ligand bound to domain I). The ligand is excluded from the calculation, and snapshots from 30–150 ns are used. Contributions shown are: a) intra-domain, b) chosen domain vs. all other domains, c) domain I vs. one other domain, d) domain II or III vs. one other domain, e) domain I and II vs. domain III and IV.

Pair distribution functions P(r) for each system have been computed as averages over the last 120 ns of simulation and are depicted in Figure S4. The first peak around 25 Å for the monomer simulations corresponds mainly to intra-domain pair distances. The second peak around 55 Å is dominated by pair distances between domains I/II and domains III/IV (cmp. Figure S3). The maximum interatomic distances D_{max}, derived from the P(r) curves, are reported in Table I. The value of D_{max} for the dimer is very close to the experimental value derived from SAXS, but the monomers have larger values by about 20 Å in the simulations. The experimental D_{max} is based on an empirical fitting of P(r), with an estimated error of ±5 Å, while the values from

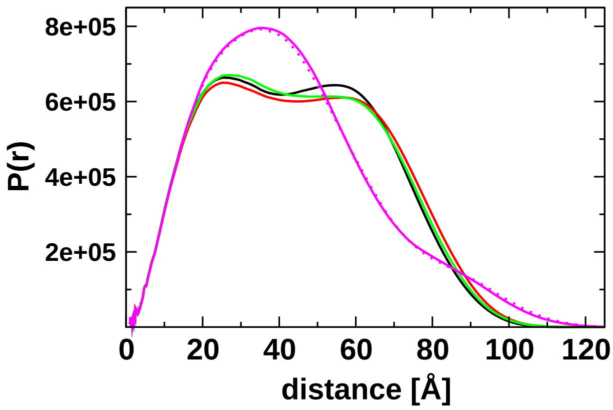

Figure S4: Pair distribution functions P(r) for the monomeric simulations bI (black), bIII (red), ub (green), and each monomer in the dimeric hEGFR simulation (solid and dotted magenta). The ligand has been omitted from the calculation.

simulation are known exactly.

With EGF bound to domain III, the P(r) curve for the receptor monomer tends to larger distances, and the second peak is shifted to slightly higher distances (see red curve in Figure S4). When EGF binds to domain I, the monomer appears to be somewhat more compact, as also suggested by R_g (see Table I). The compactness of the unbound hEGFR comes between those two, although the second peak is less well pronounced. The P(r) curve for an individual monomer extracted from the dimer simulation is clearly different, with a single main peak around 35 Å and a slight shoulder around 85 Å. This curve for an extended monomer is qualitatively similar to that found experimentally for the ErbB2 monomer (see Figure 6 of Dawson et al.[10]).

Figure S5 compares P(r) from simulation to pair distribution functions obtained from SAXS[10]. The experimental P(r) for the monomer displays a clear peak at 40–45 Å and only a weak shoulder at 65 Å while the MD simulation has a first peak at 27 Å and a much stronger shoulder visible around 55 Å. The simulation also has a longer, but little populated, tail. These discrepancies are most likely due to the contribution of oligosaccharides to the experimental curves[10], but one cannot rule out some contribution from an extended conformation in the latter (cf. Figure S4). For the dimer, both the experimental and the simulation P(r) peak at 50 Å but the simulation displays a weak shoulder at 85 Å. The tail falls off much earlier in the simulation but does extend as far as the experimental one (see D_{max} in Table I).

3 PCA analysis

The PCA (principal component analysis) method constructs a variance–covariance matrix, i.e. self–distances and distances to all other chosen atoms, as an average over the trajectory. Diagonalisation of this matrix yields eigenvectors which describe the direction of each collective motion with the associated eigenvalue being the variance, see e.g.[75]. Scalar (dot) products of PCA eigenvectors can be used to assess similarity between individual motions. For normalised vectors the value is 1 if they are identical, 0 means orthogonal, and -1 means anti-correlated. Tables S1 and S2 summarise mutual dot products for the first five modes of the bI, bIII and ub simulations. Table S1 contains dot products for simulation time 31–80 ns and table S2 for the last 50 ns (101–150 ns).

Between bI and ub simulations there is some similarity in some modes during 31–80 ns but the dot products are very different in the last 50 ns. Comparing the bIII and ub simulations

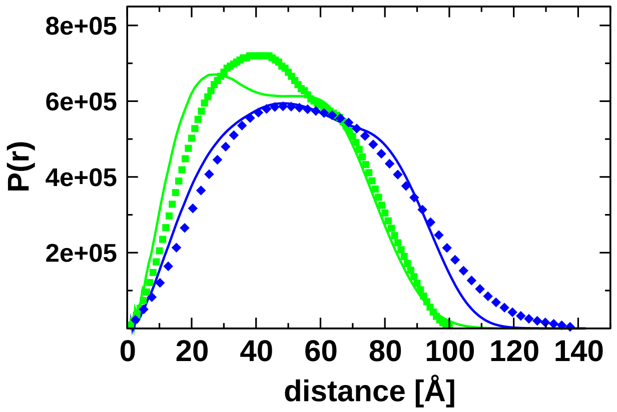

Figure S5: Pair distribution functions P(r) compared to experimental results where green squares are for the tethered unliganded monomer (cmp. Figure 2A in[10]) and the blue diamonds for the liganded dimer (cmp. Figure 5A in[10]). Solid lines are from simulation.

Table S1: Scalar products for the first 5 PCA eigenvectors for simulation time 31–80 ns.

simulation	mode no.				
ub ↓ / bI →	1	2	3	4	5
1	0.45443	-0.67694	-0.40700	-0.09109	-0.17135
2	-0.05172	0.35721	-0.75455	-0.38594	0.16148
3	0.76872	0.35699	0.24203	-0.20161	0.24796
4	-0.09371	0.20239	0.16925	-0.44158	-0.55897
5	-0.11287	-0.30533	0.19819	-0.46162	0.14305
ub ↓ / bIII →					
1	0.85765	0.00268	-0.13813	-0.31806	-0.15305
2	-0.25320	0.69443	-0.42431	-0.26041	-0.06328
3	0.07627	0.45957	0.75484	0.11632	-0.26670
4	0.15069	0.27354	0.21073	-0.24558	0.78649
5	-0.17629	-0.05767	0.11725	-0.69349	-0.27343
bIII ↓ / bI →					
1	0.48281	-0.41176	-0.56745	-0.31358	0.11103
2	0.18088	0.69435	-0.49929	0.18598	0.05789
3	0.67670	-0.03083	0.34340	0.50100	-0.03096
4	0.06949	0.13481	0.32077	-0.25758	0.76975
5	0.01970	-0.24962	-0.08818	0.29030	0.32076

Table S2: Scalar products for the first 5 PCA eigenvectors for simulation time 101–150 ns.

simulation	mode no.				
ub ↓ / bI →	1	2	3	4	5
1	-0.34554	-0.37363	0.13673	-0.18806	0.36326
2	0.07098	-0.16744	0.51876	-0.08106	-0.50563
3	0.35436	0.17695	0.48107	-0.36226	0.38486
4	0.57050	0.18946	-0.01954	0.47499	0.03691
5	-0.34045	0.73649	0.10450	0.00845	-0.10412
ub ↓ / bIII →					
1	0.38965	-0.55739	0.50714	-0.21298	0.01434
2	0.59986	0.42845	-0.21847	0.23581	-0.28524
3	-0.14992	0.50197	0.72071	0.27722	0.22863
4	-0.46877	-0.13285	0.05414	0.33218	-0.40974
5	-0.13269	0.28750	0.08493	-0.59036	-0.22584
bIII ↓ / bI →					
1	0.36262	-0.39212	-0.03071	0.36485	0.02094
2	0.51019	0.07680	0.13535	0.47752	0.36279
3	0.39968	-0.05540	0.39033	-0.61748	0.28243
4	0.21201	0.77485	0.16682	0.09871	-0.35541
5	-0.54603	0.08015	0.43410	0.27276	0.33722

of 31–80 ns there is a certain similarity of modes between the first three like-numbered modes while modes 4 and 5 appear to be swapped. Also here the last 50 ns are very different from the earlier part of the simulation except the two mode 3s. Modes from simulation bI vs. bIII show very little similarity over the course of the simulation. Therefore, the three simulations exhibit different modes after prolonged simulation, i.e. the overall movements are different from each other.

In figure S6 we have plotted the projections of the first five eigenvectors of each simulation onto the trajectory. On the right-hand panel the probabilities of the distances are shown. The first two modes display a rather broad distribution of distances with multiple peaks. This indicates that the sampling is not sufficient to cover the large amplitude modes in the simulation. The slowest mode (top row) spans a range of up to 12 Å.

We have also plotted the projection of the first eigenvector against the projection of the second eigenvector for each simulation as shown in figure S7. Thus, we receive a trajectory in two dimensions spanned by the two eigenvectors. The three simulations span different regions in space but in all cases the final position is comparably close to or overlaps with the initial coordinates. The bI simulation also displays some unoccupied regions which may indicate free energy barriers. Overall, the dynamics with respect to these two first modes appears to be different in the three simulations, however, it must be noted that eigenvectors point in different directions as shown in tables S1 and S2 and therefore direct comparison between the graphs in figure S7 is not possible.

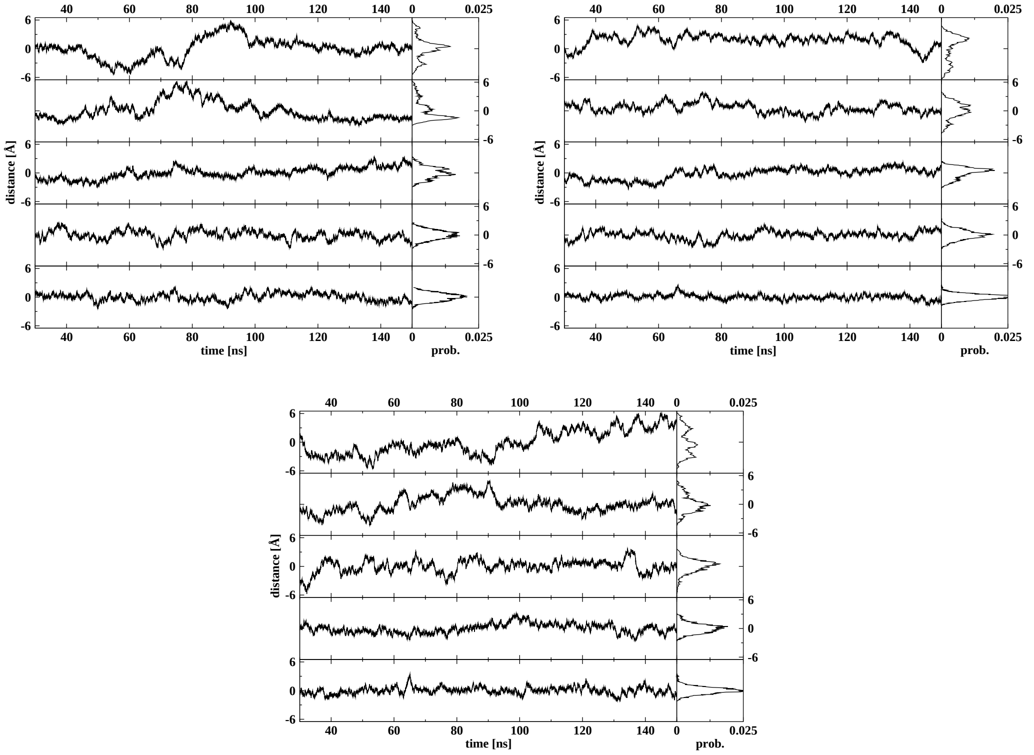

Figure S6: Projections of the first five PCA modes onto the trajectory. The right-hand panels in each subfigure show the probabilities. Top left: bI, top right: bIII, bottom: ub.

4 MM-PBSA validation

MM-PBSA calculations have been performed with CHARMM[54] c35b5 using a script developed by us[56]. To validate the correctness of our implementation we compared results obtained for a simulation of the Ras-Raf complex to previous work of Gohlke and Case who have carried out an extensive MM-PBSA analysis[35]. Here, however, we restrict ourselves to the simplified procedure in the Amber advanced tutorial number 3[76]. Ras-Raf is a central component in intra-cellular signal transduction, being part of the MAPK pathway which is activated by several receptors including EGFR[77].

MD simulations have been performed with NAMD[48] 2.6 and 2.8 using the CHARMM 22 force field for proteins[49]. Simulations were carried out with and without GTP. Parameters for GTP were constructed in analogy to ATP already present in the force field database. All simulations were carried out for 20 ns. Amber simulations were extended from the final coordinates from the tutorial. The GTP ligand was not included in those coordinates.

The final total free energies ΔG_{tot} obtained via the single trajectory approach but not including entropies are shown together with energy components in table S3. First, we observe a difference of 9 kcal/mol between Amber using their Poisson-Boltzmann (PB) solver PBSA and Baker's APBS[59]. All MM-PBSA calculations with CHARMM have been carried out using APBS. The final ΔG_{tot} is 7 kcal/mol higher than the corresponding AMBER result. Using different ion concentrations we obtain slightly higher ΔG_{tot} with the linearised PB equation similar to what was observed recently[53]. The simulation including GTP is very close to the simulations without GTP.

The differences in ΔG_{tot} may be explained in part by the usage of different force fields. Recently it was shown that simulations with the CHARMM and AMBER (Cornell et al.[78]) force fields produce similar free energies in the case of A$\rightarrow$B transition of short DNA double

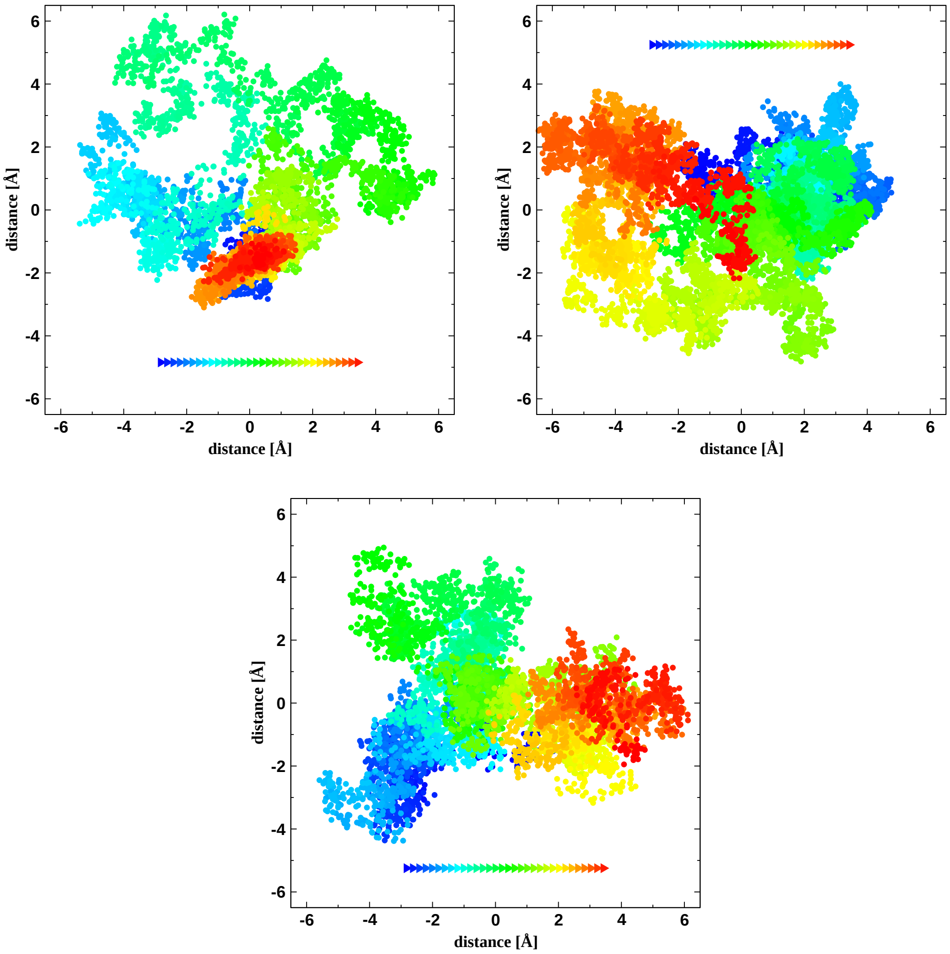

Figure S7: Projection of the PCA first mode plotted vs. the projection of the second eigenvector. Blue colours indicate earlier parts of the trajectory, red colours the later parts (see colour triangles in the graphs). Top left: bI, top right: bIII, bottom: ub.

Table S3: MM-PBSA energies obtained from simulations with Amber and CHARMM.

	Amber		CHARMM			
PB solver	PBSA	APBS	APBS	APBS	APBS	APBS
concentration	0.0 M	0.0 M	0.0 M	0.075 M	0.150 M	0.0 M[a]
ΔE_{ele}	-978.0	-978.0	-1127.5	-1127.5	-1127.5	-1098.52
ΔE_{vdW}	-64.8	-64.8	-38.8	-38.8	-38.8	-40.2
ΔE_{gas}	-1042.8	-1042.8	-1166.4	-1166.4	-1166.4	-1138.7
ΔG_{asa}	-7.2	-8.4	-7.8	-7.8	-7.8	-7.9
ΔG_{PB}	968.0	980.1	1110.2	1117.2	1118.3	1086.5
ΔG_{tot}	-82.0	-71.0	-64.0	-57.0	-55.9	-60.1

[a] Simulation with GTP.

strands[79]. The detailed energetics is different for the two force fields but the PB calculation mostly compensates for that. One other reason for the differences in ΔG_{tot} is statistics. We only ran a single simulation for each case. Multiple simulations, maybe even only short ones[80,81], could improve results and bring them closer together.

We conclude therefore that our CHARMM implementation of the MM-PBSA method is accurate and produces satisfactory results, but it is unknown which force field is more accurate and what ion concentration is appropriate. In addition, we also investigated the effect of the grid size resolution in the PB calculation on ΔG_{tot} as depicted in figure S8. As can be seen, higher grid resolutions give smaller fluctuations in the calculated free energy. In some cases lower resolution may lead to positive point-wise free energies while the highest resolution yields only negative ones. Results have been computed using the single trajectory approach. Similar observations have been made earlier[53].

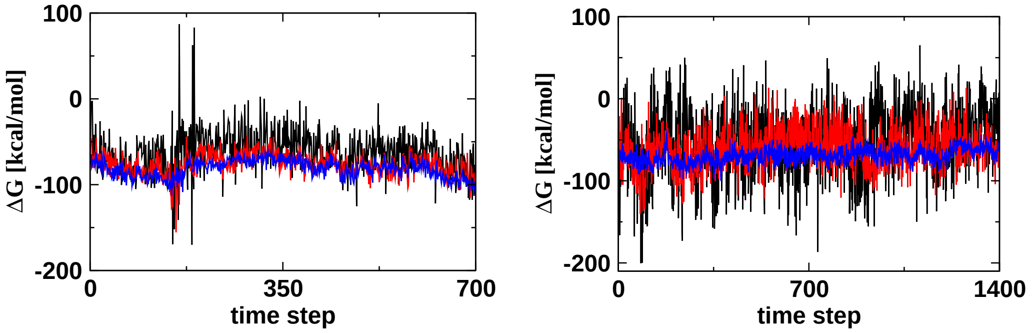

Figure S8: The effect of grid size resolution on ΔG_{tot} in MM-PBSA calculations. Left: bI simulation, right: bIII simulation. Black is 0.75 Å, red is 0.5 Å and blue is 0.3 Å resolution.

5 Entropy Calculation on Reduced System

The conformational entropy S_{MM} is difficult to obtain and there is some debate in the literature as to how to compute it[35,82]. Due to the comparatively large systems looked at here, we estimated S_{MM} through normal mode analysis obtained from 7 to 14 points along each trajectory. However, the demand in both computer time and especially memory made it necessary to obtain an estimate on a reduced system using a similar approach to that described by Kongsted and Ryde[67]. To that end, we included all residues within 8 Å of the ligand in a variable region, plus some additional residues to ensure that chains were kept as intact as possible (see Figure S9). The remainder of the binding domain (either I or III) was fixed with constraints to avoid severe distortions due to dangling bonds in the variable region, and to keep important close contact interactions. The other domains were deleted in the minimization.

6 Statistics

To estimate the correlation in our data set of simulation snapshots, we have made use of the *statistical inefficiency method*. The statistical inefficiency s can be determined through a blocking of the data whereby the variance $\sigma^2(\langle E \rangle_b)$ of increasing block sizes is compared to the variance of the total set $\sigma^2(\langle E \rangle_{\text{total}})$. $\sigma^2(\langle E \rangle_b)$ is computed as

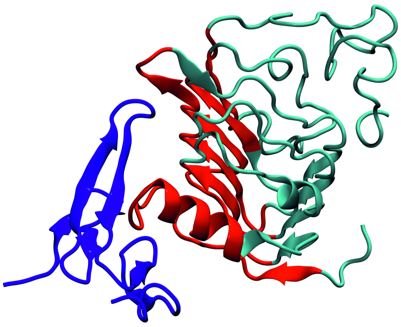

Figure S9: Residues selected for the entropy calculation, for snapshots taken from the bI simulation. The ligand (blue) is bound to domain I (cyan). The residues in red constitute the variable region to be included in the energy minimization in addition to the ligand. Residues are similarly chosen when the ligand binds to domain III.

Table S4: Statistical estimates calculated for the four independent simulations. The statistical inefficiency s is given as the number of snapshots (where snapshots are taken every 100ps or 200ps). σ and $\sigma_{\langle E\rangle}$ are in kcal/mol.

	dI (complex)			dIII (complex)			ub			EGF		
	s	σ	$\sigma_{\langle E\rangle}$	s	σ	$\sigma_{\langle E\rangle}$	s	σ	$\sigma_{\langle E\rangle}$	s	σ	$\sigma_{\langle E\rangle}$
E_{ele}	25	184.9	35.0	20	172.8	20.7	100	187.2	70.8	50	64.3	17.8
E_{vdw}	6	40.0	3.7	17	41.7	4.6	17	39.8	6.2	22	12.4	2.3
E_{bond}	5	78.1	6.6	9	74.9	6.0	1.5	74.2	3.4	1.3	22.1	1.0
E_{MM}	25	193.8	36.6	25	182.2	24.4	100	193.5	73.1	35	66.6	15.5
G_{PB}	20	169.2	28.6	25	165.6	22.1	100	178.6	67.5	40	60.6	15.0
G_{SA}	25	2.2	0.4	14	1.7	0.2	75	2.1	0.7	40	0.8	0.2
G_{noS}	15	79.5	11.6	13	73.4	7.1	4	71.7	5.4	11	22.6	2.9

$$\sigma^2(\langle E\rangle_b) = \frac{1}{n_b}\sum_{b=1}^{n_b}(\langle E\rangle_b - \langle E\rangle_{\mathsf{total}})^2 \tag{1}$$

where b is the block index of n_b blocks of t_b length and $\langle E\rangle$ the average energy. The limiting value, the statistical inefficiency,

$$s = \lim_{t_b\to\infty}\frac{t_b\sigma^2(\langle E\rangle_b)}{\sigma^2(\langle E\rangle_{\mathsf{total}})} \tag{2}$$

can be used to estimate the standard deviation of the mean energy $\sigma_{\langle E\rangle}$, the standard error, as

$$\sigma_{\langle E\rangle} \approx \sigma\sqrt{\frac{s}{N}} \tag{3}$$

where σ is the standard deviation from the energy mean and N is the total size of the data set. Table S4 lists s, σ and $\sigma_{\langle E\rangle}$ for the four independent simulations. Statistics are given for individual energy contributions.

Table S5 shows standard errors of the binding energies computed from the statistics in table S4. The listed values correspond to the three–trajectory approach, in which three inde-

Table S5: Standard errors calculated for the three trajectory approach based on data from table S4.

	dI	dIII
ΔE_{ele}	80.9	75.8
ΔE_{vdw}	7.6	8.0
ΔE_{bond}	7.5	7.0
ΔE_{MM}	83.2	78.6
ΔG_{PB}	74.9	72.6
ΔG_{SA}	0.8	0.7
ΔG_{noS}	13.2	9.4

Table S6: Statistical estimates calculated for the one trajectory approach and bI. Some data taken from table S4. s in multiples of time steps, σ and $\sigma_{\langle E \rangle}$ in kcal/mol.

	receptor			ligand			Δ^a	point–wise^b		
	s	σ	$\sigma_{\langle E \rangle}$	s	σ	$\sigma_{\langle E \rangle}$	$\sigma_{\langle E \rangle}$	s	σ	$\sigma_{\langle E \rangle}$
E_{ele}	20	145.1	24.5	40	61.7	14.8	45.2			
E_{vdw}	6	38.8	3.6	20	10.7	1.8	5.5			
E_{bond}	7	74.3	7.4	6	22.6	2.1	10.2			
E_{MM}	25	153.7	29.1	40	61.2	14.6	49.0			
G_{PB}	20	133.1	22.5	40	57.8	13.8	38.9			
G_{SA}	20	1.6	0.3	80	0.6	0.2	0.6			
G_{noS}	15	74.6	10.9	25	22.4	4.2	16.5	35	10.1	1.59

^{a} Data treated as if simulation were independent as in the three trajectory approach.
^{b} Data based on single trajectory.

pendent simulations are used. The standard error has been computed as

$$\sigma^2_{\langle \Delta \text{E} \rangle} = \sqrt{\sigma^2_{\langle \text{E} \rangle}(\text{com}) + \sigma^2_{\langle \text{E} \rangle}(\text{rec}) + \sigma^2_{\langle \text{E} \rangle}(\text{lig})} \tag{4}$$

where $\sigma_{\langle E \rangle}(\text{com})$ is the standard error for the complex, $\sigma_{\langle E \rangle}(\text{rec})$ for the receptor, and $\sigma_{\langle E \rangle}(\text{lig})$ for the ligand.

Tables S6 and S7 list s, σ and $\sigma_{\langle E \rangle}$ for the single trajectory approach for both the domain I and domain III binding cases. Individual energy components to the total free energy are shown. The last three columns denoted as "point–wise" derive from the G_{noS} computed directly from the final free energy calculated as the sum of each time step from a single trajectory. This obviously assumes dependent statistics and is shown here only for illustrative purposes. The single trajectory approach assumes that structures obtained from ensembles in the complex are representative for structures obtained from simulations of the isolated molecule and hence can be treated as if computed independently. As such also the $\sigma_{\langle E \rangle}$ must be computed as in equation (4).

Table S7: Statistical estimates calculated for the one trajectory approach and bIII. Some data taken from table S4. s in multiples of time steps, σ and $\sigma_{\langle E\rangle}$ in kcal/mol.

	receptor			ligand			Δ^a	point–wise^b		
	s	σ	$\sigma_{\langle E\rangle}$	s	σ	$\sigma_{\langle E\rangle}$	$\sigma_{\langle E\rangle}$	s	σ	$\sigma_{\langle E\rangle}$
E_{ele}	20	158.7	19.0	60	49.6	10.3	29.9			
E_{vdw}	15	39.4	4.1	50	12.1	2.3	6.6			
E_{bond}	10	71.3	6.0	4	22.3	1.2	8.6			
E_{MM}	23	168.8	21.6	55	53.1	10.5	34.2			
G_{PB}	26	152.2	20.8	60	46.6	9.7	31.8			
G_{SA}	18	1.6	0.2	40	0.7	0.1	0.3			
G_{noS}	10	70.0	5.9	10	22.0	1.9	9.4	35	8.8	1.4

^{a} Data treated as if simulation were independent as in the three trajectory approach.

^{b} Data based on single trajectory.
